# Evidence for Broadening Criteria for Atypical Depression Which May Define a Reactive Depressive Disorder

**DOI:** 10.1155/2015/575931

**Published:** 2015-07-14

**Authors:** Brett Silverstein, Jules Angst

**Affiliations:** ^1^Department of Psychology, City College of New York, New York, NY 10031, USA; ^2^Research Unit, Zurich University Psychiatric Hospital, 8032 Zurich, Switzerland

## Abstract

*Objective*. Arguing that additional symptoms should be added to the criteria for atypical depression. *Method*. Published research articles on atypical depression are reviewed. *Results*. (1) The original studies upon which the criteria for atypical depression were based cited fatigue, insomnia, pain, and loss of weight as characteristic symptoms. (2) Several studies of DSM depressive criteria found patients with atypical depression to exhibit high levels of insomnia, fatigue, and loss of appetite/weight. (3) Several studies have found atypical depression to be comorbid with headaches, bulimia, and body image issues. (4) Most probands who report atypical depression meet criteria for “somatic depression,” defined as depression associated with several of disordered eating, poor body image, headaches, fatigue, and insomnia. The gender difference in prevalence of atypical depression results from its overlap with somatic depression. Somatic depression is associated with psychosocial measures related to gender, linking it with the descriptions of atypical depression as “reactive” appearing in the studies upon which the original criteria for atypical depression were based. *Conclusion*. Insomnia, disordered eating, poor body image, and aches/pains should be added as criteria for atypical depression matching criteria for somatic depression defining a reactive depressive disorder possibly distinct from endogenous melancholic depression.

## 1. Introduction

Much research has been done on atypical depression, which appears in the DSM as a specifier for major depression [1]. Some of this research has suggested that changes be made in the criteria for atypical depression but very little of this research has dealt with the possibility of adding to the original criteria. This paper reviews four bodies of research that support the case for adding criteria. The focus here is on disordered eating and poor body image, pain, insomnia, and fatigue (although some interpret the leaden paralysis criterion as extreme fatigue) [2, 3]. Evidence is presented suggesting that the broadened criteria define a nonendogenous depressive disorder.

## 2. Argument 1: Studies That Served as the Basis of the Criteria for Atypical Depression

The existence of atypical depression grew out of work on response to antidepressant medication. Reviews of case studies [4, 5] suggested that some patients exhibiting a syndrome labelled “atypical depression” responded better to treatment with MAOIs than with tricyclic antidepressants. Based in large part on these case studies, researchers from Columbia University reviewed a series of empirical clinical studies that indicated that patients with some forms of depression responded better to MAOIs than to placebos while most patients exhibiting endogenous depression probably did not [6]. At the end of this review, the authors hypothesized the existence of an atypical depression involving lethargy, hypersomnolence, hyperphagia, rejection sensitivity, and reactivity of mood. By 1988, the Columbia group had shown that patients exhibiting these symptoms did respond more to phenelzine than to imipramine or placebo [7] and suggested that atypical depression be included in the DSM. This suggestion was first implemented in DSM-IV and atypical depression has been included as a specifier of major depressive disorder in all versions of the DSM since then.

The narrow version of the first argument for broadening the criteria is based upon details of the symptoms mentioned in the original case studies defining atypical depression and measured in the research studies reviewed by the Columbia group. In an early clinical review, West and Dally [4] summarized the characteristics of the patients with what they termed “atypical or hysterical” depression whom they found to be helped by the MAOI iproniazid. Included among the symptoms exhibited by these patients were fatigue, inability to fall asleep at night, and psychosomatic symptoms. While the authors noted that “weight loss was not a constant” (page 1492), they also wrote that, in response to the drug treatment, “weight gain might be rapid with a noticeable increase in appetite” (page 1493). No mention was made in the article of hypersomnia or overeating. In a related article on antidepressants, Sargant [5] described atypical depressives as “patients suffering from neurotic or reactive depressive illnesses or even sometimes from anxiety hysteria” (page 226). Characteristic symptoms mentioned in the article included general fatigue, pains, and considerable loss of weight. Again, no mention was made of hypersomnia (although both articles mentioned that atypical depressives often woke up fatigued after a long night's sleep) or of overeating. These authors contrasted endogenous depression with the atypical depression that responds to MAOIs. Subsequently, Paykel et al. [8] reported that a definition of atypical depression emphasizing reversed functional shift such as insomnia was related to measures of nonendogenous depression and noted that their findings demonstrated that “one of the clearest operational measures for atypical depression in the literature was confirmed… as a global measure of nonendogenous depression” (page 137).

About two decades later, Quitkin et al. [6] reviewed clinical research studies on response to MAOIs, particularly phenelzine. In a table, the authors summarized all studies comparing phenelzine and placebo in “neurotic or atypical depressives” (page 754). In the tabulated list of symptoms and characteristics that had been used to select the patients in the studies that demonstrated relatively good response to the MAOI were included difficulty falling asleep, fatigue, and atypical facial pain. Most of these studies simply described the patients as suffering from neurotic depression. Hypersomnia, overeating, and rejection sensitivity were not included in the table that served as the basis for concluding that “phenelzine is clearly effective in neurotic or atypical depressives” (page 749). Yet, at the end of the article, the authors suggested that MAO inhibitors be the first antidepressants used in treating “depressed patients with atypical features such as lethargy, hypersomnolence, hyperphagia, rejection sensitivity, and reactivity of mood” (page 759). Subsequently, the Columbia group performed their own study that found better response to phenelzine compared to imipramine or placebo among patients exhibiting depressive illness having reactive mood, plus at least one of four associated symptoms: hyperphagia, hypersomnolence, leaden feeling, and sensitivity to rejection [7]. They called for including these criteria as a separate category of atypical depression in the DSM.

In the argument presented here for broadening the criteria for atypical depression, this narrow emphasis on the particular symptoms included in these early articles but not included in the criteria for atypical depression that were created based upon these studies may be no more significant than a more general point. That is, in the original summary of research on MAOI response [6], the Columbia group noted that many of the studies they reviewed were done prior to DSM-III without detailed operationalization of the patients treated. They described most of the studies as dealing with patients that were not endogenous but were “inadequately characterized” (page 751), usually only described as exhibiting neurotic depression. They noted that the criteria they suggested for patients best treated with MAOIs were based on clinical impression.

This is not to suggest that the criteria were simply wrong. Scores of subsequent studies have demonstrated the usefulness of the concept of atypical depression (although it may not necessarily be so atypical) including more recent reviews of the preferential response of atypical depression to MAOIs [9]. The work of the Columbia group and some others that have done much of the research has been extremely useful. Later work done by several investigators has discussed changes that might be made to the original criteria, such as eliminating the necessity for exhibiting mood reactivity [10], according primacy to rejection sensitivity [11], or eliminating the trait features of mood reactivity and rejection sensitivity while focusing on the state features of hypersomnia and overeating and perhaps leaden paralysis [12]. But, with the exception of some studies of the possible role played by panic [13] and of age of onset and chronicity [14, 15], only one study [16] to our knowledge has suggested adding symptoms to the criteria. That is, a set of criteria based upon clinical judgement of some similarities between studies of response to MAOIs has, for the most part, been reified such that they are either applied as originally suggested or reordered in some way. That many of the early cases and research studies considered atypical depression to be a form of nonendogenous reactive depression and explicitly included insomnia, pain, weight loss, and fatigue has been ignored and left out of subsequent research.

## 3. Argument 2: Studies of the Prevalence of the Individual Symptoms of Atypical Depression

This section deals with symptoms that are included in the current DSM criteria for major depression. Some studies have measured the extent to which respondents who exhibit syndromes related to atypical depression report the various depressive criteria.

Seemüller et al. [17] measured symptoms reported by depressed patients on the Hamilton Scale for Depression (HAMD-21) in nine psychiatric hospitals in Germany. 127 patients met criteria for atypical depression. It should be noted that some of the criteria were approximated. For rejection sensitivity, Seemüller et al. used a measure of irritability and for leaden paralysis a measure of heaviness in legs. Of the patients with atypical depression, 63% reported early insomnia, 72% reported middle insomnia, 38% reported loss of weight, 52% reported loss of appetite, and 93% reported somatic symptoms, which were comprised of heaviness of limbs, aches, or fatigue.

An analysis of correlates of atypical depression was performed on data from the Children in the Community study, a longitudinal study of a systematic sample of households that had children aged 1–10 in 1977 living in two counties in New York State [16]. The data were from 1987, when respondents were 11–22. Respondents were categorized as exhibiting atypical depression if either the respondent or the respondent's mother reported that the respondent had at least a 2-week period of depressed mood but not a 2-week period of anhedonia and exhibited at least two of the following: excessive eating or weight gain during the period, excessive sleep or long naps for 2 weeks, leaden paralysis (which may have been difficult for mothers to infer from behavior) for at least 2 weeks, and scoring at least one standard deviation above the mean on a scale of items measuring rejection sensitivity. The proportion of atypical respondents who generally took at least 30 minutes to fall asleep was 42% and 63% characterized themselves as exhibiting insomnia.

The other studies described in this section did not use the criteria for atypical depression related to mood reactivity, leaden paralysis, and enduring rejection sensitivity. Angst et al. [2] described symptoms reported by respondents characterized as exhibiting atypical depression in the Zurich study, a sample representative of the Swiss canton of Zurich. 37 respondents met criteria for major depressive disorder and reported at least two of overeating, oversleeping, and excessive physical fatigue. Of these, 51% reported increased appetite compared to 54% who reported loss of appetite, 43% reported weight gain compared to 38% who reported weight loss, and 89% reported hypersomnia compared to 78% who reported insomnia. Pollitt and Young [18] found little difference between early and late insomnia among patients who responded to MAOIs versus those who did not.

Also of interest are studies that have reported the frequencies of depressive criteria exhibited by respondents who were categorized as belonging in subtypes resembling atypical depression found in latent class analyses (LCAs). Sullivan et al. [19] found one subtype of depression in a LCA of data from the National Comorbidity Survey characterized by “nearly universal presence of major depression and the preponderance of classical depressive symptoms (i.e. weight and appetite loss, insomnia, psychomotor retardation, anergia, and poor concentration)” (page 1400) that they labelled “severe typical.” The “severe atypical” subtype was characterized by the presence of major depression and appetite increase and weight gain along with sleep problems. Of the respondents in the severe atypical class, 39% exhibited appetite decrease, 40% exhibited weight decrease, 87% exhibited insomnia which was higher than the 54% of respondents in the severe atypical class who reported hypersomnia, and 92% of respondents in both the severe atypical and the severe typical classes exhibited tiredness/lack of energy.

Based upon a LCA of data from 488 respondents in the Netherlands Study of Depression and Anxiety, Lamers et al. [20] designated one class of respondents as exhibiting severe typical depression and another as exhibiting severe atypical depression. The severe typical class was characterized by decreased appetite, weight loss, and insomnia, whereas the severe atypical class was characterized by “increased appetite and weight gain” (page 2086). Separate LCAs were performed two years apart. Among respondents belonging to the severe atypical class in both analyses, the probability of fatigue was extremely high, the probability of weight loss or decreased appetite was extremely low, and the probability of insomnia was distinctly greater than the probability of hypersomnia. LCAs performed on data from the Zurich study by Rodgers et al. [21] arrived at similar results which will not be described here as the same database was analyzed in the Angst et al. [2] study described above.

Some of the studies reviewed in this section did not apply strict DSM criteria. Nonetheless, the syndromes resembling atypical depression described in all of these studies clearly involve vegetative symptoms in addition to those commonly referred to as “reversed.” The studies based on latent class analyses reported low levels of weight loss and decreased appetite but they reported very high levels of insomnia and fatigue. The studies that applied modified criteria for atypical depression to a large sample of hospitalized patients [17] and to a representative sample in the Zurich study [2] found that small majorities of people meeting these criteria reported loss of appetite and large majorities reported insomnia (fatigue was one of the criteria in the Angst et al. study and subsumed under a larger category of somatic symptoms in the Seemüller et al. study).

The characterization of atypical depression as involving reversed vegetative symptoms has been extremely useful in the recognition of the distinct nature of the syndrome. Most of the early research that led to the inclusion of atypical depression in the DSM and much of the later research that validated its existence were based on comparisons with typical depression. If the criteria for atypical depression used in these studies had included symptoms similar (but as discussed below, not necessarily identical) to typical depression, the results of the comparisons between the two subtypes would have been much weaker. The distinct character of atypical depression might never have been recognized.

It was this contrast with typical depression, apparent from the beginning even in the use of the term “atypical” in the name, that led to conceptualizing the vegetative symptoms of atypical depression as only those that are reversed. But possibly appetite loss and certainly insomnia are common among people with atypical depression. Does it make sense to eliminate them from the criteria simply because they resemble symptoms of typical depression? It is possible that the insomnia exhibited by people with typical depression arises from endogenous distortions in neurotransmitters while the insomnia exhibited by people with atypical depression arises from nonendogenous anxiety. It is also possible that the loss of appetite/weight exhibited by people with typical depression also arises from endogenous distortions in neurotransmitters while the loss of appetite/weight reported by several people who exhibit atypical depression is related to nonendogenous, psychosocially based disordered eating, as discussed below. Whether or not these hypotheses eventually receive support, should the criteria for a disorder be chosen based upon characteristics that another disorder does not have? Following this logic that seems to have been used to eliminate appetite loss and insomnia from criteria for atypical depression would result in eliminating depression from criteria for bipolar disorder because it also characterizes major depression.

## 4. Argument 3: Studies of Symptoms/Disorders That Are Comorbid with Atypical Depression

The symptoms focused on in this paper that are not included in current criteria for major depression have been reported to be comorbid with atypical depression in several studies, although the exact prevalence of the comorbid symptoms and disorders found in these studies varies. This is likely due to the variation between these studies in the samples analyzed and the definitions of atypical depression utilized.

In the Zurich study described above [2], 68% of respondents exhibiting major depression with atypical features reported symptoms characteristic of a broad definition of migraine headache. The analysis of data from the Children in the Community study [16] found that 47% of atypical respondents reported headaches/stomachaches. These results confirm the conclusion made by Davidson et al. [22] as early as 1985 based upon an analysis of patients with chronic pain that there exists an important link between atypical depression and chronic pain.

Several studies have reported a relationship between bulimia/binge eating and atypical depression. Kendler et al. [23] performed a latent class analysis applied to 14 disaggregated DSM-III-R symptoms for major depression reported by members of 1029 female-female twin pairs. They found high lifetime rates of bulimia (19%) in the group defined by this LCA as exhibiting atypical depression.

Among respondents in the Zurich study who met criteria for major depressive disorder and reported at least two of overeating, oversleeping, and excessive physical fatigue, eight percent reported bulimia and 19% reported binge eating [2]. Posternak and Zimmerman [24] evaluated 579 psychiatric outpatients with a current diagnosis of major depressive disorder for the presence of atypical features. Of those meeting DSM-IV criteria for atypical depression, six percent met criteria for bulimia. Perugi et al. [25] examined in a semistructured format 107 consecutive patients who met DSM-IV criteria for a major depressive episode with atypical features. They found that 18% of patients who met the DSM-IV criteria for atypical depression also met criteria for bulimia nervosa.

Several studies have also found a relationship between atypical depression and problems with body image. Nierenberg et al. [26] investigated 350 consecutive outpatient subjects with major depression who entered an antidepressant treatment study. They found a higher rate of lifetime and current Body Dysmorphic Disorder (BDD) in subjects with DSM-III-R atypical depression than in those with nonatypical depression (14% compared to 5%). In a study of 86 major depressive patients with atypical features as defined by the DSM, 42% were found to have BDD [25]. The Posternak and Zimmerman [24] study of psychiatric outpatients described above also found that those with atypical depression exhibited higher rates of BDD than those with typical depression (7% versus 2%). The analysis of the Children in the Community study found that 68% of respondents with atypical depression reported experiencing fear of fat [16].

It has been noted that rather than reflecting the concomitance of two distinct disorders, in some cases, comorbidity may actually reflect multiple manifestations of a single disorder [27]. Thus, one explanation of these patterns of alleged comorbidity is that pain, bulimia/binge eating, and body image issues may belong with current criteria for atypical depression as symptoms of a single disorder. The last two symptoms (in combination with the loss of appetite/weight discussed above that might result from intentional food restriction) suggest the possibility that the appetite symptoms of atypical depression may be related to a larger pattern of disordered eating.

## 5. Argument 4: Studies of Somatic Depression

The studies reviewed above suggest a possible role played in the disorder currently diagnosed as atypical depression by fatigue (as some have already acknowledged), insomnia, disordered eating, poor body image, and aches/pain. This combination indicates a similarity with somatic depression, which has been generally defined in studies as depression plus three of (1) fatigue, (2) insomnia, (3) disordered eating (either bingeing, purging, or intentional food restriction), (4) poor body image/preference for thinness, and (5) frequent severe headaches [28]. High levels of somatic depression among women have been hypothesized to account for the gender difference in the prevalence of depression [29].

In an analysis of data from a representative sample in the Zurich study [30], atypical depression was defined as meeting DSM-III criteria for depression and reporting three of the following: overeating, oversleeping, leaden paralysis, mood reactivity, and rejection sensitivity. Half of the male and the vast majority of the larger group of female respondents (83%) who met these criteria also met criteria for somatic depression. Fewer than 1/3 of the male and 1/6 of the female respondents with somatic depression met criteria for atypical depression. Thus, it might be said that atypical depression is subsumed under the more inclusive category of somatic depression, particularly for females. Therefore, it is possible that some of the findings from studies of atypical depression have been due to its overlap with somatic depression. For example, in the Zurich study, the higher prevalence among women compared to men of atypical depression was found to be due almost entirely to its overlap with somatic depression. Women exhibited 10.2% higher lifetime prevalence of any depression than did men. This total was made up of women exhibiting five percent higher prevalence of depression that met criteria for both atypical and somatic depression, 4.1% higher prevalence of somatic depression without atypical depression, and only 0.2% higher prevalence than men of atypical depression without somatic depression. That is, high levels of somatic depression among women accounted for the gender difference in depressive prevalence whereas there was little gender difference in atypical depression that did not meet criteria for somatic depression. The higher prevalence of depression among women compared to men has also been found to be almost entirely due to the higher prevalence among women of somatic depression in several other large epidemiologic databases such as the National Comorbidity Survey, the National Comorbidity Survey-Replication, and the Epidemiologic Catchment Area Study [31].

Several published studies (reviewed in [31]) have found that the high prevalence of somatic depression among females is associated with measures of psychosocial issues related to gender roles. These results are relevant here because they suggest that somatic depression is a reactive depression similar to the early descriptions of atypical depression as a form of reactive depression. Women who reported feeling that they had been limited by responses to their gender, by scoring high in studies measuring their beliefs that men lead better lives than women, or in studies measuring their beliefs that their fathers showed a preference for males, or in studies measuring their beliefs that their mothers felt limited by being female, reported high prevalence of somatic depression. Some of the studies found women's reports of somatic depression to be related to reports made by their parents of attitudes toward gender that might be expected to bother many contemporary women who aspire to more equal treatment than was historically given to females. These included fathers' reports of believing in the superiority of males and mothers' reports of having been limited by their gender. These findings support the hypothesis that somatic depression may grow out of reactions to psychosocial influences related to a lack of acceptance that may be experienced by many contemporary women who aspire to achieve in areas once considered to be the province of men. In that sense, it is possible that these results may be linked to both the symptom of rejection sensitivity included in criteria for atypical depression and the early descriptions of atypical depression as a form of reactive depression, not an endogenous depression. On this note, in the Star*∗*D study, both somatic depression [31] and atypical depression [32] responded relatively poorly to treatment with antidepressants such as citalopram. That somatic depression can result from psychosocial issues that may be particularly bothersome to contemporary “nontraditional” women may help to explain the high prevalence of depression that has been seen during recent decades [31].

In summary, the results of all of the studies reviewed here suggest that the original criteria for atypical depression should be broadened. Fatigue should be recognized separately from leaden paralysis, insomnia, headaches, and disordered eating, including bingeing as well as intentional food restriction, and poor body image/fear of fat should be added. Further studies are called for as to whether the addition of these symptoms defines a nonendogenous reactive disorder (somatic depression) that is not simply a specifier of major depression but perhaps a separate nonendogenous disorder distinct from endogenous/melancholic depression.
